# Hepatic encephalopathy: a critical current review

**DOI:** 10.1007/s12072-017-9812-3

**Published:** 2017-08-02

**Authors:** Anna Hadjihambi, Natalia Arias, Mohammed Sheikh, Rajiv Jalan

**Affiliations:** 1 Division of Medicine, UCL Medical School, Royal Free Hospital, UCL Institute for Liver and Digestive Health, Rowland Hill Street, London, NW3 2PF UK; 2 Centre for Cardiovascular and Metabolic Neuroscience, Neuroscience, Physiology and Pharmacology, University College London, London, WC1E 6BT UK; 3 INEUROPA (Instituto de Neurociencias del Principado de Asturias), Oviedo, Spain

**Keywords:** Hepatic encephalopathy, Treatment, Classification, Pathogenesis

## Abstract

Hepatic encephalopathy (HE) is a serious neuropsychiatric complication of cirrhosis and/or porto-systemic shunting. The clinical symptoms are widely variable, extending from subtle impairment in mental state to coma. The utility of categorizing the severity of HE accurately and efficiently serves not only to provide practical functional information about the current clinical status of the patient but also gives valuable prognostic information. In the past 20–30 years, there has been rapid progress in understanding the pathophysiological basis of HE; however, the lack of direct correlation between pathogenic factors and the severity of HE make it difficult to select appropriate therapy for HE patients. In this review, we will discuss the classification system and its limitations, the neuropsychometric assessments and their challenges, as well as the present knowledge on the pathophysiological mechanisms. Despite the many prevalent hypotheses around the pathogenesis of the disease, most treatments focus on targeting and lowering the accumulation of ammonia as well as inflammation. However, treatment of minimal HE remains a huge unmet need and a big concerted effort is needed to better define this condition to allow the development of new therapies. We review the currently available therapies and future approaches to treat HE as well as the scientific and clinical data that support their effectiveness.

## Introduction

Hepatic encephalopathy (HE) is defined as “brain dysfunction caused by liver insufficiency and/or porto-systemic shunting manifesting as a wide spectrum of neurological or psychiatric abnormalities ranging from subclinical alterations to coma” [1]. The recognition that liver disease and in particular jaundice could be associated with mood and behavioral disturbances can be traced back to the father of Western medicine, Hippocrates (460–371 BC) [2]. However, it was not until experimental work in the late nineteenth and the twentieth century that pathophysiological mechanisms of this relationship started to unravel, instigating a shift leading to the understanding that behavioral changes are an integral consequence of chronic liver insufficiency and disease.

## Classification of hepatic encephalopathy

The clinical presentation of HE may be highly variable constituting, a myriad of signs and symptoms ranging from defects in cognition, personality and intellect to altered conscious state and impaired neuromuscular function such as asterixis and hyperreflexia. The heterogeneous manifestations of HE vary not only between patients but also longitudinally for an individual patient. Furthermore, important observations that cirrhotic patients who appear clinically “normal” could also have defects on electroencephalography [3] and neuropsychometric testing [4] have led to the concept of minimal HE (mHE). In order to reconcile short comings and difficulties in accurately defining and classifying the severity of HE, in 1998 the World Organisation of Gastroenterology introduced a multiaxial definition of HE [5], which categorized HE according to; (1) etiology (Type A—Acute Liver failure, Type B—Portosystemic bypass without intrinsic liver disease, Type C—Cirrhosis), (2) severity (minimal or West Haven Grade 1–4 [Table 1]), (3) time course (episodic, recurrent, persistent), and (4) precipitated versus spontaneous. The EASL-AASLD consensus modified this in 2014 but kept the main elements of this classification [1].

**Table 1:** West Haven criteria for grading severity of HE

Grade	Clinical features
I	Trivial lack of awareness
	Euphoria or anxiety
	Shortened attention span
	Impairment of addition or subtraction
II	Lethargy or apathy
	Personality change
	Disorientation for time
	Inappropriate behavior
III	Somnolence to semi-stupor
	Confusion
	Gross disorientation
IV	Coma

The West Haven Criteria categorizes HE into 4 stages based solely on clinical criteria, and is often used arbitrarily and subjectively by clinicians in routine practice rather than considering all manifestations in a particular stage [6]. It has good functionality in distinguishing those patients at the lower and higher ends of the scale, but the main limitation lies in accurate identification and discrimination of grade 1 HE from those who have no HE and those with mHE due to significant inter- and intra-observer inconsistency [7]. The Hepatic Encephalopathy Scoring Algorithm (HESA) [8] combines clinical and neuropsychological assessment as a means of improving the sensitivity of grading and has shown promise in clinical trials [9]. The Clinical Hepatic Encephalopathy Staging Scale (CHESS) [10] was developed as a newer method, and grades severity of HE in a linear fashion from 1 to 9, but is not widely used. Grade II HE is more readily discriminated using disorientation and asterixis as markers, which has led to a proposal by the International Society for Hepatic Encephalopathy and Nitrogen Metabolism (ISHEN) to term HE Grade ≥II as Overt HE (OHE), whereas Grade 1 and mHE may be classed as Covert HE (CHE) [7]. OHE and CHE can be viewed as tangible points in a paradigm that considers the spectrum of neurocognitive impairment in cirrhosis as a continuum rather than categorical [11]. The term CHE is limited, being in essence an umbrella term. A study of 132 cirrhotics demonstrated CHE to be heterogeneous syndrome requiring a combination of clinical and neuropsychometric indicators for diagnosis [12]. In a recent prospective study, Thomsen et al. [13] showed that patients with Grade 1 HE were clinically, pathophysiologically and prognostically distinct from those with mHE, suggesting that lumping Grade 1 HE and mHE together under the term CHE is potentially flawed.

The utility of categorizing the severity of HE accurately and efficiently serves not only to provide practical functional information about the current clinical status of the patient but gives valuable prognostic information and presents objective standards for research and trials involving HE. The current challenge thus is of first clarifying what is “normal”, as an absence of HE does not necessarily equal absence of neuropsychometric abnormality [14], and then determining optimal methods by which early HE can be consistently and accurately distinguished. Montagnese et al. [14] advocate the advantages of considering an individual’s lifelong neuropsychometric performance and co-morbidity in determining whether any abnormality equates to HE.

## Neuropsychometric assessment

Although termed minimal, mHE is not trivial, having been associated with a multitude of deleterious effects on quality of life [15], including sleep disturbance [16], falls [17], ability to drive [18] and impacts on employment ability, with knock-on effects on associated socio-economic status [19]. The occurrence of mHE predicts the onset of OHE [20] and adversely affects survival [21]. Several methods are available to diagnose mHE, including common tests such as Psychometric Hepatic Encephalopathy Score (PHES), Critical Flicker Frequency (CFF) and Electroencephalogram (EEG); less widely-used tests include the scan test, Continuous Reaction Time (CRT) Test, Inhibitory Control Test (ICT) and repeatable battery for the assessment of neuropsychological status (RBANS). A significant issue, however, is just the moderate concordance between differing methodologies, [22] which is likely indicative of the tests reflecting different pathologies [23], and thus they should be considered as complementary rather than equivalent [14]. Given the detrimental impact of mHE, there is an urgent need to translate diagnostic criteria and tests into pragmatic everyday clinical practice [24].

Assessing the degree to which cognitive dysfunction is caused by hepatic insufficiency can be challenging, as not only can a multitude of potential concurrent factors such as infection, renal failure, drugs, or pre-existing cognitive/psychiatric disorders contribute to and exacerbate HE, but causes of underlying liver disease such as alcohol [25], obesity [26] and Hepatitis C [27] may also lead to impairment in cognitive ability. This distinction is important in order to allow identification and correct individual treatment of all mechanisms causing altered mental status. HE is observed in a one third of all patients admitted to hospital with acute decompensation [28]. The presence of HE is predictive of worse survival in both patients with and without Acute-On-Chronic Liver Failure (ACLF) [29]. Isolated HE in acute decompensation is different to the ACLF, in that it is associated with older age, inactive drinkers and not fundamentally related to the severity of underlying liver disease, highlighting the predisposition of other insults to the brain (ageing and alcohol) in the development of HE [29]. In ACLF, HE is more frequent in younger patients with more severe liver failure, infection and evidence of systemic inflammation (white cell count and C-reactive protein). Cognitive impairment in cirrhosis is strongly associated with bacterial infection; a prospective study demonstrated cognitive impairment (overt or subclinical) in 42% of cirrhotics without infection, in 79% with infection and in 90% with sepsis and systemic inflammatory response [30]. It is proposed that HE in ACLF is clinically distinct from that in acute decompensation driven by diverse pathological mechanisms in each entity [31], underscoring the multifactorial pathogenesis of this condition. More studies are therefore required for the better understanding of each condition and mechanism of development of HE.

## Pathophysiological mechanisms of HE

**Fig. 1:**
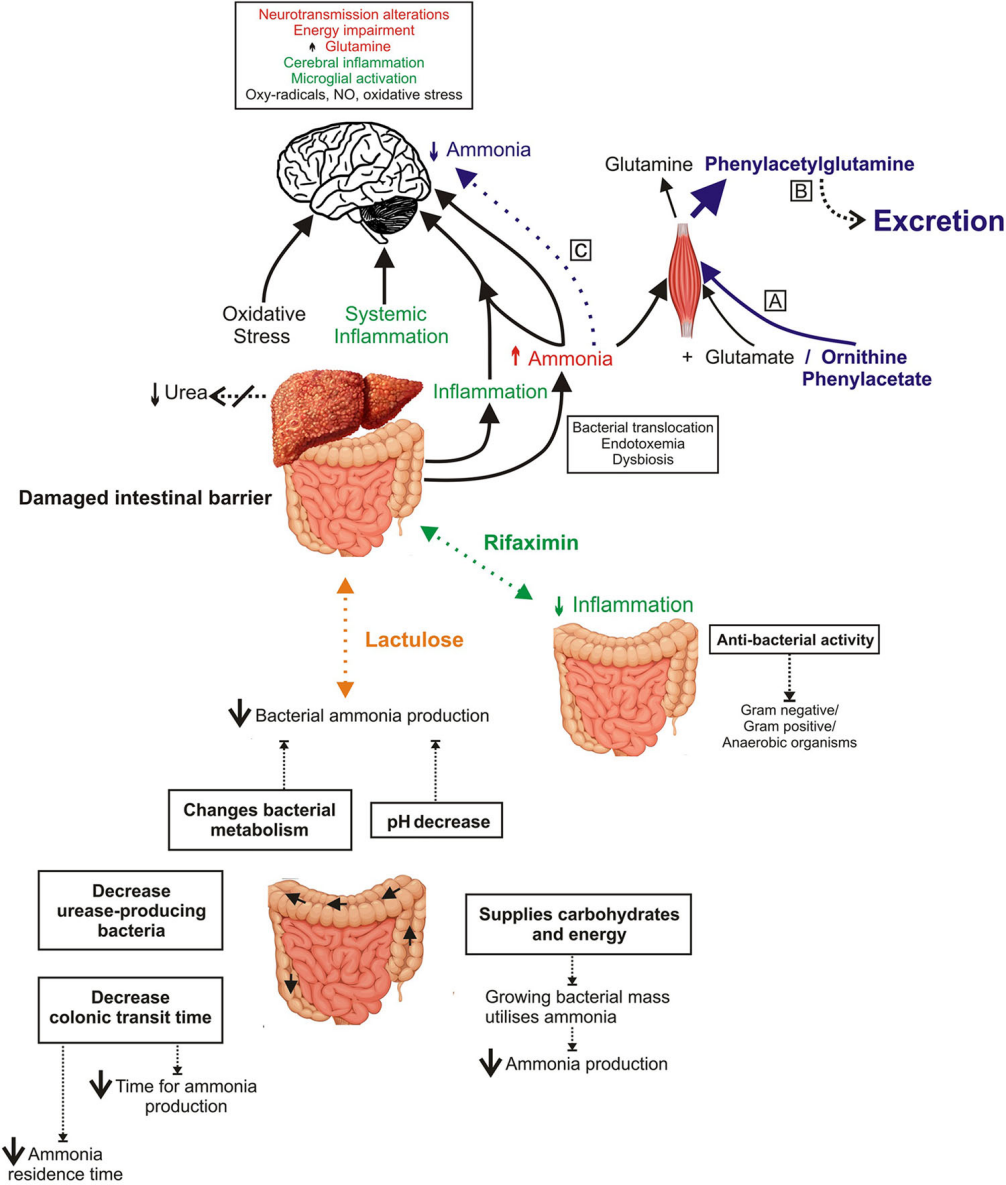
Factors contributing to the pathogenesis of HE and treatment mechanisms. Factors contributing to the pathogenesis of HE with emphasis on the interorgan effects of ammonia and inflammation arising due to liver disease. The mechanism of action of ornithine phenylacetate (OP), which acts in lowering hyperammonemia through the production of the L-ornithine induced glutamine is depicted in *blue* (*A*). Glutamine in then converted to phenylacetylglutamine in the kidney followed by its eventual excretion (*B*). As a result, this increases the update of available ammonia for the production of glutamine, which consequently lowers the systemic and brain ammonia levels (*C*). The mechanism of rifaxamin is indicated by *green arrows*. Lactulose, in *orange*, effectively targets the gut and lowers bacterial ammonia production. *OP* ornithine phenylacetate, *NO* nitric oxide, *NH*_*3*_ ammonia, *NH*_*4*_^*+*^ ammonium ions

HE occurs due to a combination of distinct pathophysiological mechanisms such as inflammation [32], oxidative stress [33], impaired blood–brain barrier (BBB) permeability, neurotoxins, impaired energy metabolism of the brain [34] and more (Fig. 1). Moreover, it is assumed that HE is caused by similar mechanisms both in mHE and overt states of the syndrome. However, recent clinical and experimental data suggest that there are discrepancies between studies indicating that HE is likely to be a heterogenous entity. In this section, we will try to assess the current limitations in our understanding of the pathophysiology of HE.

### Brain edema and energy metabolism

In acute liver failure (ALF), the deficiency of energy metabolism associated with brain edema has been fully described. This energy dysfunction is thought to be due to a compromised tricarboxylic acid cycle enzyme, α-ketoglutarate dehydrogenase activity, limited anaplerotic flux and capacity of astrocytes to detoxify ammonium by glutamine synthesis, increased lactate synthesis as well as mitochondrial permeability transition induced by oxidative/nitrosative stress [34, 35]. At later stages of the disease, several mechanisms have been proposed where the circulating ammonia can increase glutamine, which could secondarily impact energy metabolism through an initial osmotic stress, while changes in the glutamate–glutamine cycle will follow. Additionally, brain edema is life threatening and in these patients brainstem herniation and death can occur [36]. However, instead of a severe edema seen in ALF, mild edema is seen in chronic liver failure (CLF), which correlates with lower and more variable ammonia concentrations [37]. Finally, the presence of edema is described infrequently in ACLF patients where it is only present in 4–8% of patients [38].

**Fig. 2:**
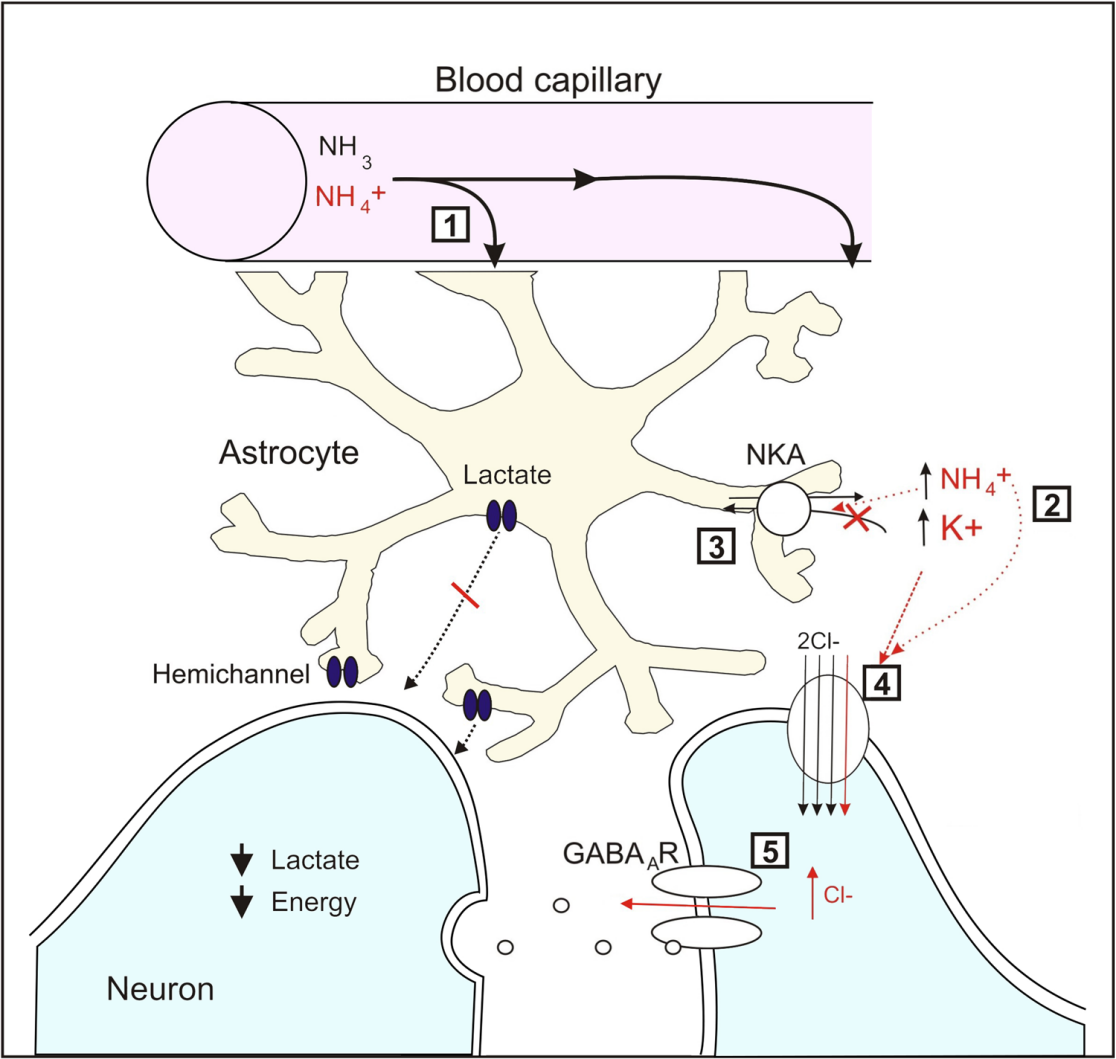
Mechanism of action of ammonia on astrocytic and neuronal dysfunction modified by Hadjihambi et al. [108]. (*1*) Astrocytes entrap plasma ammonia and act as a sink for ammonia detoxification in brain by way of the enzyme glutamine synthetase. (*2*) This short-circuits potassium buffering, resulting in increased [K^+^]_o_. (*3*) Increase in [NH_4_^+^]_o_ and [K^+^]_o_ stimulate Na^+^–K^+^ ATPase(NKA) activity. (*4*) The excess [NH_4_^+^]_o_ and [K^+^]_o_ promotes overactivation of neuronal NKCC1, which is the principal neuronal chloride importer. (*5*) This results in an increase in [Cl^−^] leading to neuronal E_GABA_ depolarization and therefore neuronal disinhibition. A recent study has also revealed impairment of hemichannel function and lactate release, due to hyperammonemia, which implies limited energy supply to the already compromised neurons

In line with this, a recent ex vivo study in 6 weeks bile-duct ligated (BDL) rats showed that a rise in lactate (1.7 fold) and not glutamine is an important player in the pathogenesis of brain edema in CLF [39]. In an apparently contradictory study, Hadjihambi et al. [40] showed a decrease in extracellular lactate in the cerebral cortex, due to hemichannel dysfunction, (Fig. 2), which suggests that the total brain lactate increase reported by previous studies would be due to its intracellular accumulation. Interestingly, Rackayova et al. [41] reported no significant elevation of lactate in rats 8 weeks following BDL, which is a very severe model of HE. These observations are very important as lactate does not only act as an osmolyte but is also an essential energy metabolite, reflecting potential cerebral energy impairment in patients with HE.

### Neurotransmission alterations

Another pathogenic mechanism in HE associated with energy disturbances is the alteration in neurotransmission systems, such as the glutamatergic and GABA-ergic systems, resulting in neuronal disinhibition (mechanism described in Fig. 2). However, recent studies in a model of 8-week BDL rats [41] have shown no changes in GABA concentrations but observed a decrease of glutamate and aspartate while glutamine concentrations were elevated. Therefore, contrary to the traditional approaches, these changes could be explained as the result of ammonium detoxification driven by increased glutamine synthesis from glutamate in astrocytes, without necessarily affecting neurotransmission at late stages of the disease. However, a more thorough investigation of mild models of CLF needs to be performed with more precise techniques for neurotransmitter examination.

### Bile acids

The presence of huge concentrations of bile acids was recently shown to be present in the cerebrospinal fluid (CSF) of cirrhotic patients with HE [42]. In animal models, it has been shown that rats with acute galactosamine-induced liver failure exhibit regional cerebral edema, indicating that the BBB had lost, at least in part, its barrier function [43]. In a model of BDL, a significant increase in circulating bile acids and a compromise in the integrity of the BBB were found. In the light of these studies, increases in serum bile acids are not just a feature of biliary disorders. Spillover of bile acids into the circulation are also observed during ALF [44], ACLF [44], and non-alcoholic steatohepatitis [45], and even in the CSF of cirrhotic patients. Therefore, the direct role of bilirubin or bile acids in the development of HE should also be reconsidered.

### Manganese accumulation

Manganese deposits have been described as a cofactor in the development of HE. The observation that the reduction in brightness of the basal ganglia observed on magnetic resonance imaging rapidly improves after liver transplantation [46] may be supportive of the manganese deposition hypothesis. However, the observation that, in occupational manganese exposure, the resolution in cerebral pallidal T1 hyperintensity is much slower [47] suggests that this is a more complex issue.

### Inflammation

It is important to highlight that brain cell damage is not only a consequence of the development of HE but also a contributing factor. Under these circumstances, it has been shown that astroglia releases TNF-α, followed by a release of glutamate while also activating microglia [48]. Microglia activation is usually followed by proliferation and release of pro-inflammatory cytokines such as TNF-α, IL-1β and IL-6 [48], while there is strong evidence that this inflammatory state can lead to neuronal death in vitro and in vivo [49, 50]. It is well known that this inflammatory state can be triggered from systemic inflammation underlying the gut–liver–brain axis alteration, which includes direct effects of systemic pro-inflammatory molecules in the brain, recruitment of monocytes after microglial activation and altered permeability of the BBB [51].

There is evidence based on both animal and human studies showing that high ammonia induces HE only if systemic inflammatory response syndrome (SIRS) is present [43, 52]. Thus, it is widely accepted that sepsis is able to trigger HE in cirrhotic patients as a result of altered nitrogen metabolism and also by releasing pro-inflammatory mediators [53]. In 2000, Rolando and colleagues suggested that, in ALF patients, the presence of SIRS resulted in a poorer neurological outcome [31]. Furthermore, in ACLF, sepsis is an important precipitating factor for decompensating liver failure and HE in previously stable patients with cirrhosis [54], indicating the importance of inflammation in the pathogenesis of the disease. The CANONIC study also demonstrated the clear role of systemic inflammation in patients with advanced HE which also correlated with mortality [55].

## Treatment

The approach to the treatment of HE depends on its severity. Ammonia remains the main target and is aimed at reducing the production of ammonia and maximizing the body’s removal of ammonia from the bloodstream. However, ammonia metabolism is complex and is regulated in various organs such as the liver, muscles, kidneys and brain. Therefore, the drugs used to treat HE need to be well understood and undergo regulated clinical trials to maximize their effectiveness. Additionally, lack of treatment for other precipitating factors involved in the development of HE, such as oxidative stress, inflammation or other cerebral alterations, is a major limiting step in the treatment area. The first priority of treatment depends on actively identifying and treating all potential precipitating causes, such as infection, electrolyte disturbance, dehydration, etc., while managing the complications of encephalopathy. Finally, recurrence of HE needs to be addressed when the patient recovers from the acute episode. Despite the subtle nature of minimal and episodic HE, it can have significant effects on a patient’s daily living. Unfortunately, at this time, only OHE is routinely treated and overall medical treatment options for HE are limited. Finally, a more personalized treatment will have to be developed for patients, without focusing only on the stage of HE but also considering the disease responsible and its history. Some of the most current and emerging treatments for HE are discussed in the following section and depicted in Table 2.

**Table 2:** Current treatments for HE, suggested prescribed dose and effectiveness

Treatment	Example	Dose	Effect
Non-absorbable disaccharides	Lactulose	30–80 g/day	Decrease plasma ammonia
	Lactitol	5–360 days	
Antibiotics	Rifaximin	550 mg twice daily	Decrease serum levels of ammonia and bacterial translocation
Amino acids	l-ornithine-l-aspartate (LOLA)	0.25/(kg bodyweight/day)	Decrease serum ammonia levels
Ammonia scavenger	Ornithine phenylacetate (under experimental conditions)	Phase II trials (final dose yet to be determined)	Decrease plasma ammonia and neuroinflammation
	Glycerol phenylacetate (HPN-100)	6 mL bid	Decrease plasma ammonia
	Polyethylene glycol (PEG)	0.25 g/L orally	Decrease plasma ammonia
Albumin dialysis	MARS (Molecular Adsorbent Recirculating System)	Albumin dialysis	Targets inflammation and reserved for specialists centers only
Radiological Interventions	Occlusion of spontaneous shunts	Radiology	Reducing ammonia by targeting portosystemic shunting
Probiotics	Various	Various	Decrease intestinal pH
			Decrease blood ammonia levels
			Alter microbiome composition
Nutritional therapy	Changes in diet	35–45 kcal/g	Unknown
		1.2–1.5 g/kg protein/day	
Branched-chain amino acids (BCAAs)	Various		Unclear
Experimental	Bromocriptine	Various	Increase dopamine neurotransmission
	Minocycline	100 mg/daily	Decrease plasma and cerebrospinal fluid ammonia levels
	Ibuprofen	Unknown	Targets neuroinflammation
	Sildenafil	25–50 mg	Reduces neuroinflammation and restores cognition
	Indomethacin	0.5 mg/kg	Targets neurosteroids and Neuroinflammation
	Ro15-4513	Unknown	Increase neurological scores and EEG tracing Decrease bleeding
	Fecal microbiota transplantation (FMT)	4 FMT	Targets intestinal dysbiosis

### Non-absorbable disaccharides and polyethylene glycol

Lactulose and to a lesser extent lactitol are standard treatments that are aimed at reducing the amount of ammonia absorbed into the blood stream. One of the actions of lactulose is to create a hyperosmolar environment and act as a laxative preventing efficient absorption of ammonia by the colon (Fig. 1). Despite the lack of evidence for the use of lactulose on patients with acute HE [56], a recent meta-analysis showed that non-absorbable disaccharides have beneficial effects in the treatment and prevention of HE. Furthermore, other benefits include reduction in serious liver-related morbidities and all-cause mortality [57]. Studies comparing lactulose to control are lacking and, therefore, no comparative studies exist to strongly validate the use of lactulose for the treatment of hyperammonemia [58]. Additionally, one study showed that nearly 50% of HE recurrence was related to either no adherence or inappropriate dosing [59]. There is, however, a danger for overuse of lactulose leading to complications, such as dehydration, aspiration, hypernatremia and perianal skin irritation. This can lead to difficulties during liver transplantation and can cause malnutrition in patients [59] or even precipitate HE. Despite the limited evidence of efficacy, lactulose is widely used in clinical practice. While some studies have shown benefits in primary prophylaxis with lactulose [60], current guidelines do not recommend the routine use of lactulose as primary prophylaxis for HE [61]. Additionally, Rahimi et al. [61], performed a randomized clinical trial comparing polyethylene glycol (PEG) and lactulose treatments in patients with cirrhosis admitted to the hospital for HE. The results of this study revealed that PEG led to more rapid HE resolution than standard therapy, suggesting that PEG may be superior to standard lactulose therapy in patients with cirrhosis hospitalized for acute HE. However, more studies are required to establish the effectiveness of this compound.

### Antibiotics

Antibiotics are divided into two classes: absorbable and non-absorbable antibiotics. Within the absorbable category, neomycin has been previously used extensively in the management of acute, but not chronic HE. A study has demonstrated that neomycin is as effective as lactulose in 33 patients [62], although, in another randomized study by Strauss, neomycin was shown to be no better than a placebo in patients with HE [63]. Patients who are maintained on chronic neomycin must have periodic evaluation for potential side effects while more clinical trials are required to evaluate the effectiveness of this antibiotic. In the poorly absorbable antibiotics category, rifaximin is the most commonly used antibiotic for the treatment of HE and has also been used in a number of trials [64]. A meta-analysis by Wu et al. [65] has shown that rifaximin is as effective and potentially better than non-absorbable disaccharides at treating HE. A double-blind, randomized study with 120 patients revealed a significant decrease in OHE when both treatments, lactulose and rifaximin, were used in combination rather than when lactulose treatment was used alone. Length of hospital stay was also significantly decreased [66]. The data clearly demonstrate that patients tolerate rifaximin better and that best results are achieved when it is coupled with lactulose. Moreover, a cohort study of 299 patients revealed a reduced risk of hospitalization involving HE over a 6-month period with the use of rifaximin [67]. Despite this, further research is required to support the use of rifaximin alone since limitations such as lack of placebo represent the current studies [68]. The only significant complication observed so far with this antibiotic involved two unique cases of *Clostridium difficile* infection [69].

### l-ornithine and L-aspartate

l-Ornithine and L-aspartate (LOLA) are substrates for the urea cycle and can increase urea production in periportal hepatocytes. They also activate glutamine production by activating glutamine synthetase in perivenous hepatocytes and skeletal muscles. Studies have indicated that LOLA use was ineffective in reducing the ammonia concentration and the severity of HE in ALF patients [70]. However, in patients with CLF, an improvement in recurrent bouts of HE was shown, while the treatment was well tolerated and was shown to be superior to placebo [71]. Furthermore, although LOLA initially lowers blood ammonia levels, even in end-stage liver disease, its effects appear to be temporary as a rebound hyperammonemia is sometimes observed on cessation of the drug [72]. Further research is required in determining amount, duration and dosage of this treatment.

### Ornithine phenylacetate

Drug treatment with ornithine phenylacetate (OP) focuses on the formation of glutamate and the removal of glutamine. l-Ornithine is active in the synthesis of glutamate. OP stimulates glutamine synthetase activity in peripheral organs [73]. The consequent increase in glutamine synthesis results in a net decrease of plasma ammonia. Finally, in order to prevent the ‘rebound effect’ of glutaminase, glutamine is conjugated with phenylacetate to form phenylacetylglutamine, a molecule that cannot be metabolized and is harmlessly excreted in the urine [74]. OP successfully prevents increases in arterial ammonia while evidence exists from animal models suggesting a significant decrease in extracellular brain ammonia, preventing intracranial hypertension and improving the mental state (mechanism of action summarized in Fig. 1) [75]. OP is currently in Phase II trials and a small open-label study has shown that the administration of OP was safe and resulted in a marked reduction in ammonia concentration [73]. However, a recent randomized clinical trial in 38 consecutive cirrhotic patients, enrolled within 24 h of an upper gastrointestinal bleed, indicated that OP appeared well tolerated although failed to significantly decrease plasma ammonia at the given doses (10 g/day) [76]. In a large clinical trial in about 230 patients with OHE, preliminary data shows that the administration of OCR-002 results in a dose-dependent significant reduction in ammonia and an improvement in the severity of HE (unpublished data; www.oceratherapeutics.com)

### Glycerol phenylbutyrate (HPN-100)

Glycerol phenylbutyrate (GPB) provides an alternative pathway for ammonia removal and waste nitrogen excretion in the form of phenylacetyl glutamine. This results in a lower net blood glutamine and, therefore, ammonia production from the action of glutaminase. In a randomized, double-blind Phase IIb study, effectiveness of this drug was shown in cirrhotic patients with evidence of a reduction of ammonia and a reduction in both the recurrence of HE and re-hospitalization [77]. However, this effect was lost when co-administered with rifaximin. This suggests its potential usefulness in secondary prophylaxis of HE. However, more clinical trials are needed to establish the effectiveness of this drug as a treatment of HE.

### Albumin administration and dialysis

Albumin can be beneficial to HE patients because it has anti-oxidant properties and is able to scavenge reactive oxygen species. However, studies on albumin administration in patients with stage II or higher HE was ineffective in resolving HE severity, ammonia levels, oxidative stress markers or cytokines, but it prolonged survival [77]. It was also found that albumin dialysis using the Molecular Adsorbent Recirculating System (MARS) in patients with HE showed a quicker improvement than in patients not treated with this device [78]. MARS also achieved decreases in plasma bilirubin, ammonia and creatinine concentrations as well as improved portal pressure in ACLF [79]. However, there was no significant improvement in severe HE [80]. Further studies are required for conclusions to be drawn and to evaluate the efficacy of this treatment.

### Portosystemic shunt occlusion

Large portosystemic shunts bypass the liver, resulting in hyperammonemia and HE. While embolization of these shunts was shown to resolve HE immediately, it is only effective in the presence of residual liver function. A retrospective study evaluating patients with recurrent HE who achieved complete occlusion of portosystemic shunts compared to patients who did not undergo the procedure showed some benefits in embolization [81]. In patients with end-stage liver disease, this treatment not only is less effective but can also be deleterious, and therefore patients need to be selected carefully [82]. An endovascular approach to manage shunts is minimally invasive and may involve occlusion of the shunt with coils or plugs, or, if associated with varices, a balloon-occlusion retrograde trans-venous obliteration technique may be used to obliterate both [83].

### AST-120

AST-120 involves the use of orally administered microspherical carbon, which exhibits a selective adsorbent profile, adsorbing small molecules such as ammonia. A study on rodents with CLF treated with AST-120 indicated decreased arterial ammonia levels, normalized brain water content and locomotor activity, but did not demonstrate an effect on systemic oxidative stress [84]. In contrast, a study by Bajaj et al. [85] failed to report an improvement in CHE. A phase II trial (Ocera Therapeutics (2014) https://clinicaltrials.gov/ct2/show/NCT00867698) showed a comparable efficacy to that of lactulose in patients with mHE, with fewer side effects, although more studies are required for the further development of this treatment.

### Probiotics

Probiotic therapies attempt to adapt the gut environment. This is an attempt to limit the amount of ammonia produced in the colon. Patients treated with probiotics often show lowering of blood ammonia and less severe HE, although no significance has been shown [86]. Additionally, a systematic review of nine randomized control trials (RCTs) concluded that probiotics were associated with improvement in mHE, prophylaxis of OHE and reduction in severe adverse events [87]. The use of probiotics in secondary prophylaxis was also evaluated in an open-label RCT, dividing 235 patients into probiotic, lactulose or no therapy groups [88]. Both lactulose and probiotics were more effective than no treatment, although there was no significant difference between them. Despite the lack of significance in this data, there seems to be no adverse consequence of using these treatments. Finally, a recent meta-analysis has shown that, overall, the use of probiotics was more effective in decreasing hospitalization rates, improving mHE and preventing progression to OHE than placebo, while the use of probiotics did not affect mortality rates. However, given the huge variability in the type and composition of probiotics and the lack of validation studies, its use is not routinely recommended for patients with HE.

### Nutrition and branched-chain amino acids

Malnutrition can lead to a paradoxical increase in ammonia and decreased survival by influencing protein turnover [89], increasing susceptibility to infections, impairing immunocompetence [90] and inducing malabsorption [91]. It is believed that maintaining muscle mass in patients is important, since it has the ability to remove ammonia from circulation, while patients administered with enough protein observed a beneficial effect in the management of hyperammonemia and HE [92].

Administration of branched-chain amino acids (BCAAs) is believed to help improve nutrition and may be effective in HE treatment. However, BCAA have paradoxically been seen to increase blood ammonia levels [93]. It is recommended that BCAA be used only by patients who are severely protein-intolerant. If given, BCAA should be administered orally, as opposed to intravenous treatment which may result in lower gut glutaminase activity [56]. A meta-analysis of eight trials conducted by Gluud et al. [94] concluded that oral BCAA improved manifestations of HE but showed no effect on overall mortality or nutrition status. Once again, there is not enough evidence through clinical trials for these treatments to be used consistently with confidence, and they usually just act as complimentary treatments.

As increased GABA-ergic neurotransmission has been implicated in neuronal inhibition associated with HE, the efficacy of utilizing benzodiazepine receptor antagonists to treat HE has been evaluated in several RCTs. A meta-analysis of 6 RCTs with a total of 641 patients found that flumazenil (benzodiazepine receptor antagonist) treatment versus placebo resulted in significant clinical improvement of HE (odds ratio = 6.15; 95% CI 4.0–9.5; *p* < 0.001), but the trials were limited by very short follow-up period, a maximum 72 h in 2 trials and less in the rest [95.] A Cochrane review analyzed 13 randomized trials (2 of which were included in the previous meta-analysis) with a total of 805 patients comparing flumazenil versus placebo [96.] Flumazenil was associated with only short-term improvement in HE (risk difference 0.28; 95% CI 0.20–0.37) with no significant effects on recovery from HE or survival. Short-term benefit of flumazenil with the absence of prolonged effects is not unsurprising given its short elimination half-life (0.7–1.3 h), and thus it is not currently advocated for routine clinical use to treat HE.

l-Carnitine plays a key role in mitochondrial energy metabolism by facilitating carriage of long-chain fatty acids from the cytoplasm into the mitochondrial matrix and in portocaval-shunted rats shown to have a protective effect against hyperammonaemia [97]. A meta-analysis of 7 RCTs, including 660 patients with HE grade ranging from subclinical to grade 3, found l-Carnitine to reduce ammonia levels, with associated improvement in HE [98]. However, all 7 RCTs were performed at a single center and were of small to moderate size. Although the results are encouraging, further larger multi-center studies are required before routine use of l-carnitine for HE can be advocated.

Treatment with plasma exchange can modulate systemic inflammation in ALF, leading to improved outcomes, and it is associated with improvement in cerebral oxygen and improved HE [99]. However, in the largest trial of plasma exchange for ALF, no significant difference was noted in intracranial pressure between the two groups, likely because of under-powering as only 16 patients had intracranial pressure measured, with no further details about improvement in HE [100].

### Future approaches

Whereas liver transplantation is the most effective treatment available, it is not always an option. There are a few treatments still in the experimental phase which hold future promise for the therapy of HE (Table 3) such as Minocycline, which reduces microglial cell activation in cerebral insult and results in a reduction of brain edema as well as plasma and CSF ammonia levels. Ibuprofen has been shown to restore the function of the glutamate–nitric oxide–cyclic guanine monophosphate (cGMP) pathway in cerebral cortex and learning ability in portocaval-shunted rats. Phosphodiesterase-5 inhibitors such as sildenafil can also be beneficial in chronic HE. Indomethacin, a potent inhibitor of 3a-hydroxysteroid dehydrogenase, a key enzyme responsible for the synthesis of allopregnanolone (ALLO) and tetradehydrodeoxycorticosterone (THDOC) (potent selective positive allosteric modulators of the GABA_A_ receptors), has been shown to attenuate brain production of ALLO and THDOC and to improve activity in animals with CLF and mild HE [101]. It is important to note that non-steroidal anti-inflammatories such as ibuprofen and indomethacin would need to be used extremely cautiously in cirrhotics due to their effects on other systems such as renal function. Benzodiazepine inverse agonists such as Ro15-4513 have also been shown to be effective in the treatment of HE in animal models of acute [102] or chronic liver failure [103]. Finally, a phase II study is currently underway investigating whether fecal microbiota transplantation can reverse HE in cirrhotic patients with episodic HE despite maintenance therapy with lactulose and/or rifaximin. Additionally, a small study performed recently reported beneficial effects of this treatment in the management of HE [104]. Despite the new and interesting treatments arising, it is essential for the current treatments used in clinics to be well understood and also to undergo regulated studies before their use on patients.

**Table 3:** Future approaches with potential for clinical application

Compound	Target	Potential indication	Study	Comments
Non-steroidal anti-inflammatory	Glutamate–NO–cGMP pathway	Cirrhosis	[105]	Ibuprofen
Minocycline	Microglial cell activation	Acute liver failure	[106]	Can cause hepatotoxicity
Phosphodiesterase inhibitors	Glutamate–NO–cGMP pathway	Cirrhosis	[107]	Sildenafil
Indomethacin	GABA(A) receptor complex	Cirrhosis	[101]	Targets THDOC and ALLO
Ro15-4513	GABA(A) receptor	Acute or chronic liver failure	[103]	Benzodiazepine inverse agoinist
Fecal microbiota Transplantation	Gut: enteric bacteria flora	Cirrhosis	[104]	Trial: http://clinicaltrials.gov/show/NCT02255617

## Conclusions

In the past 20–30 years, there has been rapid progress in understanding the pathophysiological basis of HE. Many new strategies are addressing the issue of hyperammonemia but the lack of direct correlation between ammonia levels and the severity of HE makes it difficult to select patients for liver transplantation. The results of the Phase IIb study of OP are awaited, as it will provide the first evidence of the role of ammonia in patients with an acute episode of HE on the background of CLF. The recently defined entity of ACLF and the identification that this entity is distinct from acute decompensation has huge implications for the selection of HE patients for clinical trials. Finally, treatment of mHE remains a huge unmet need and a huge concerted effort is needed to better define this condition to allow the development of new therapies.

## Abbreviations

ACLF: Acute-On-Chronic Liver Failure
ALF: Acute liver failure
ALLO: Allopregnanolone
BBB: Blood–brain barrier
BCAAs: Branched-chain amino acids
BDL: Bile-duct ligated
cGMP: Cyclic guanosine monophosphate
CFF: Critical Flicker Frequency
CHE: Covert hepatic encephalopathy
CHESS: Clinical Hepatic Encephalopathy Staging Scale
CLF: Chronic liver failure
CRT: Continuous Reaction Time
CSF: Cerebrospinal fluid
EEG: Electroencephalogram
FMT: Fecal microbiota transplantation
GPB: Glycerol phenylbutyrate
HE: Hepatic encephalopathy
HESA: Hepatic Encephalopathy Scoring Algorithm
ICT: Inhibitory Control Test
ISHEN: Society for Hepatic Encephalopathy and Nitrogen Metabolism
LOLA: l-Ornithine and L-aspartate
MARS: Molecular Adsorbent Recirculating System
mHE: Minimal hepatic encephalopathy
NKA: Na^+^–K^+^ ATPase
OHE: Overt hepatic encephalopathy
OP: Ornithine phenylacetate
PEG: Polyethylene glycol
PHES: Psychometric Hepatic Encephalopathy Score
RBANS: Repeatable battery for the assessment of neuropsychological status
RCTs: Randomized controlled trials
SIRS: Systemic inflammatory response syndrome
THDOC: Tetradehydrodeoxycorticosterone
